# Peer Mentors for People with Advanced Cancer: Lessons Learnt from Recruiting and Training Peer Mentors for a Feasibility Randomized Controlled Trial

**DOI:** 10.1007/s13187-020-01692-7

**Published:** 2020-01-28

**Authors:** Catherine Walshe, Diane Roberts, Lynn Calman, Lynda Appleton, Robert Croft, Guillermo Perez Algorta, Suzanne Skevington, Mari Lloyd-Williams, Gunn Grande

**Affiliations:** 1 International Observatory on End of Life Care, Division of Health Research, Lancaster University, Bailrigg, Lancaster UK; 2 Division of Nursing, Midwifery and Social Work, Manchester University, Manchester, UK; 3 Macmillan Survivorship Research Group, School of Health Sciences, Southampton University, Southampton, UK; 4 Clatterbridge Cancer Centre NHS Foundation Trust, Wirral, UK; 5 Liverpool, UK; 6 Division of Health Research, Lancaster University, Lancaster, UK; 7 Manchester Centre for Health Psychology, School of Psychological Sciences, Manchester University, Manchester, UK; 8 APSCSG, Institute of Population and Health Sciences, Liverpool, UK

**Keywords:** Cancer, Palliative care, Peer Mentor, Volunteer, Recruitment, Feasibility study

## Abstract

Peer mentors may offer distinctive forms of support to people with advanced cancer. Whilst peer mentor programmes are known, little is understood about recruiting and training peer mentors to support those with advanced cancer. The purpose of this study is to determine the feasibility of recruiting and training peer mentors for a novel peer mentor intervention to promote well-being in people with advanced cancer. Feasibility study testing proactive introduction to a trained peer mentor for 12 weeks in the context of a randomized controlled two-arm trial and nested qualitative process evaluation was used. Peer mentors have/had cancer, recruited via an open call. Two-day training included a new bespoke module on coping with cancer. Descriptive recruitment and training data were captured, supplemented by qualitative interviews, analysed thematically. Forty-eight people expressed interest, mostly female (69%), with breast cancer (32%), and recruited via social media (49%). Twelve people completed training, with attrition often due to availability or mentors’ own health; many had advanced cancer themselves. They wanted to ‘give something back’, but also formed supportive bonds with fellow mentors. It is feasible to recruit and train people with lived experience of cancer to be peer mentors, but those with particular characteristics may predominate. Broad social media based recruitment may have merit in widening the pool of potential peer mentors.

## Introduction

*There also is likely to be an untapped community of cancer survivors who would like to support others’ recovery* [36].

Recent commentary highlighted the potential importance of peer support for those with cancer but also the challenge of recruiting peer mentors [34, 36]. Such recruitment for studies or support programmes is rarely explicitly explored. The aim in this paper therefore is to present novel data on the process and experience of recruiting peer mentors in the context of a feasibility randomized controlled trial of peer mentoring for people with advanced cancer.

People with advanced cancer can experience negative emotions, stress, depression and anxiety [25, 33]. However, it is possible to develop coping strategies such as pragmatism, self-awareness and reliance on others that can enable psychological well-being to be managed or regained [37, 46]. Whilst information about coping with cancer from healthcare professionals is valued, people with cancer often want to learn about developing effective coping strategies from their peers [2, 5, 46].

Peer support is increasingly common, and there is emerging evidence of beneficial effects for both mentors and mentees [18, 34, 35, 38, 45]. Peer support, within a healthcare context is defined as ‘the provision of emotional, appraisal, and informational assistance by a created social network member who possesses experiential knowledge of a specific behaviour or stressor and similar characteristics as the target population, to address a health-related issue of a potentially or actually stressed focal person’ [10]. Peer support involves people using their shared personal experience to provide knowledge, social interaction, emotional assistance or practical help, often in a way that is mutually beneficial [31]. There are important differences in the content and style of peer interactions in comparison to those with health professionals and emotional support through this route is particularly valued [12, 14]. Peer support is different because the source of support is a similar person with relevant experience, and health policy recognizes the importance of such support [32]. Peers may have insights into emotional and practical aspects of living with cancer that health and social care professionals may not have and their input congruent with theoretical perspectives such as helper therapy principles and theories of social support processes and buffering effects [24, 30].

Whilst peer support can be available for those with cancer, there appear to be few studies of peer mentoring specifically for those with advanced cancer [4, 11, 13, 15, 21, 26, 28]. However, the needs of those with advanced cancer are distinct because of their health status [1], and there may be differences in their desire for, experience, and impact of different forms of peer support. A recent review of peer mentoring for people with advanced cancer found that only two studies exclusively focused on this patient group but that people with advanced cancer were active and major users of peer support. Few robust trials of peer support exist [45].

Qualitative research with those with advanced cancer, and their informal carers, found a strong preference for peer support as an intervention to enable coping with advanced cancer [37, 46]. We therefore conducted a feasibility randomized controlled trial of a peer mentor intervention for people with advanced cancer. Central to the success of this trial was the recruitment and training of those willing to be peer mentors to those with advanced cancer. Little is known about the process of recruiting and training peer mentors, yet it is critically important to the planned intervention. This paper adds to this knowledge through describing their recruitment, training, challenges and lessons learnt. This learning should also have utility for those finding and training peer mentors within and outside the context of a research study.

## Methods

### Study Aims and Design

The overall aim of the study was to determine the feasibility of delivering and investigating a novel peer mentor intervention to promote and maintain psychological well-being in people with advanced cancer using a randomized controlled trial design. The specific aims related to recruiting and training peer mentors for the study were:

i. Understanding from where and how to recruit peer mentor participants to support people with advanced cancer
ii. Exploring why people volunteer to be peer mentors to people with advanced cancer
iii. Understanding issues of retention and attrition of peer mentors through their training and their perceptions on such training

This paper reports on the important aspects of recruiting and training peer mentors for the study. The design of the study was a feasibility randomized controlled trial, with embedded qualitative process evaluation. Data presented here are captured from the trial database but also qualitative interviews with those who completed training as peer mentors.

### Population

Peer mentors who possessed experiential knowledge of coping with a cancer diagnosis and treatment [10] were recruited. They need not have advanced cancer themselves but having so did not exclude them from this role. The inclusion and exclusion criteria for potential participants are specified in Table 1.

**Table 1:** Inclusion and exclusion criteria for peer mentors

Peer mentor inclusion criteria	Peer mentor exclusion criteria
1. Experience of living with cancer but at least 6 months post diagnosis	1. Aged under 18
2. Able to commit to 6 months of volunteering and have 2+ hours a week available for peer mentoring	2. Live outside the geographical area of the project
3. Qualitative demonstration of empathy, compassion and open and nondidactic communication skills	3. Insufficient fluency in written and spoken English
4. Satisfactory completion of project specific training	
5. Disclosure and Barring Service (police) clearance for working with vulnerable people	

### Sampling and Recruitment

An open call for potential peer mentors was appropriate, as there is no available sampling frame of those who meet the criteria, and recruiting via clinicians is not advocated [39]. This call was widely disseminated via a number of channels including posters at two participating cancer centres in public areas, via internal communication networks such as newsletters, by social media platforms such as Twitter and Facebook, and a press release which resulted in articles placed in some local newspapers and two interviews with investigators on local radio. In addition, information was placed with local volunteer bureaux and the volunteering website (http://www.do-it.org.uk/). Potential participants were requested to contact the study team to have an initial discussion about the research and the peer mentoring element of this work and to determine initial eligibility for the study. If they remained interested in the study, written study information was provided, and they were invited to attend an initial training day.

### Peer Mentor Training

There is little evidence on the optimum length, mode or content of training programmes for peer mentors or other similar patient facing volunteers. Recent reviews have variously reported no trials in the area of volunteer training for those in palliative care roles [16] and little information on the training of peer supporters [9] and variation in duration, location, format, learning strategies employed and occupation of trainer and content of training reported in included studies [44]. Training typically takes place on more than 1 day, with a varied focus on organizational content (understanding the service to be provided, personal safety, lone working arrangements, safeguarding, etc.), service provision and facilitation skills (communication skills, managing emotions, etc.) and preferred content (patient navigation, etc.) [6–8, 19, 22].

For this trial, we chose to run a 2-day training programme, offered flexibly to suit those who volunteered as potential peer mentors. Training days were not consecutive but agreed with participants after day 1 training. We used established training materials for peer mentors and other volunteers working with those with cancer used by Macmillan Cancer Support as our basic training [42]. This was, with permission, adapted for this project, with acknowledgement given to Macmillan Cancer Support for the underpinning materials. These materials formed the basis for the first day of the training and incorporated information on personal safety, lone working, safeguarding, home/community visiting, appropriate boundaries and communication skills. The second day of training reinforced communication skills training, with a bespoke module on coping with advanced cancer. The materials for this bespoke module were generated from our underpinning qualitative research and focused on the coping mechanisms elicited from the analysis of interviews of 28 people with advanced cancer and their family carers [46]. All training materials were delivered in a face-to-face group setting. Activities were designed to be interactive and to promote discussion and learning with and from other training participants. Participants were provided with a folder of written materials summarizing the learning activities to support the face-to-face training. Applications were made during the training period to the Disclosure and Barring Service (DBS – criminal records checks) for potential peer mentors.

### Entering the Study as a Peer Mentor

At the completion of the training and with return of an appropriate DBS check, people were assessed by the study team for their suitability as peer mentors. Eligibility criteria (box 1) included an informal appraisal of their empathic communication skills demonstrated during the training period and assessed by the trainers and an enhanced understanding of the peer mentor role. At this point, for those who were assessed as suitable and who wished to progress into the trial as a peer mentor, written consent to participate in the trial was obtained, and they signed a study-specific volunteer agreement specifying shared rights and responsibilities on behalf of both the study team (e.g. to provide support and supervision) and the peer mentor (e.g. to maintain confidentiality).

### Data Collection and Analysis

Data were collected throughout the study on a study database to track those who enquired about peer mentoring, including how they heard about the opportunity, brief biographical details and their progress through training. Qualitative face-to-face or telephone interviews were conducted towards the end of the study, inviting mentors to reflect on their recruitment and training. Interviews were conducted by DR (qualitative researcher) and CW (researcher and palliative care nurse). Written consent was obtained, and interviews digitally audio recorded and transcribed. Data were managed using NVivo11. Inductive coding was followed by identification of core themes. Data were initially stored and backed up at the University of Manchester using password protected central institutional filestores. Data were shared with the principal investigator using Lancaster Box which is an enterprise cloud storage solution. It uses high-grade encryption to secure data both in transit and rest.

## Results

Data are presented here on recruitment of peer mentors, their characteristics, motivations for becoming peer mentors and their perceptions on training and its timing (from peer mentor interviews *n* = 7).

### Peer Mentor Recruitment

**Fig. 1:**
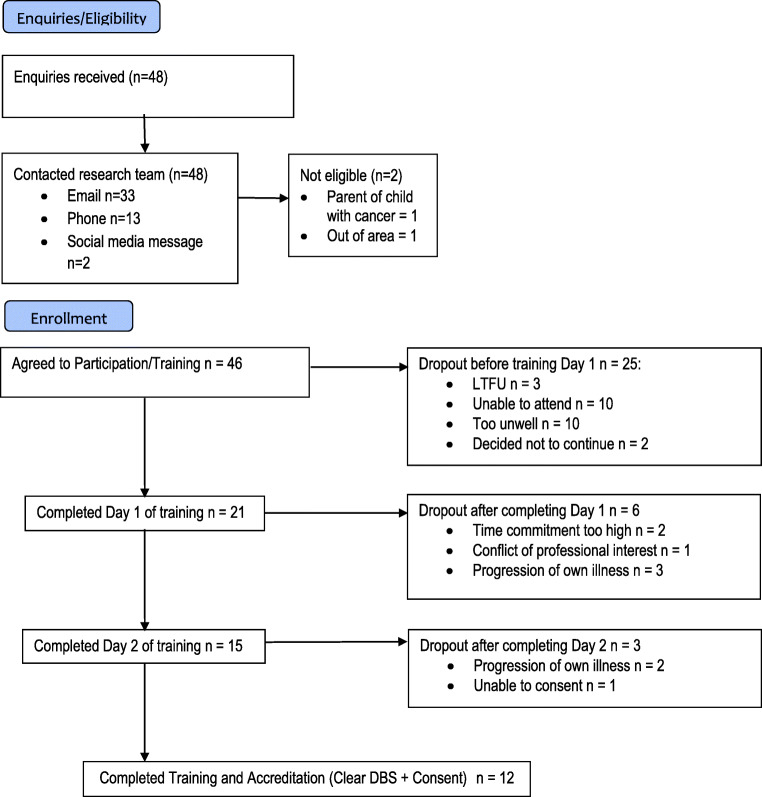
Peer mentor recruitment and training flow diagram

Over an 8-month period (2016–17), 48 people enquired about becoming a peer mentor. Their flow through the study is displayed in Fig. 1.

Whilst enquiries about the peer mentor opportunity continued to arrive for 15 months, most enquiries were received within the first 3–4 months of the opportunity being initially advertised. Details of recruitment source for peer mentors who enquired, attended training and completed training are shown in Table 2.

**Table 2:** Characteristics of those who enquired about, and subsequently trained to be, peer mentors

	Enquirer characteristics		Characteristics of trained peer mentors		
		Number (%)	Attended day 1	Attended day 2	Accredited
**Associated with:**	Cancer centre A	25 (52%)	13 (62%)	10 (67%)	8 (67%)
	Cancer centre B	23 (48%)	8 (38%)	5 (33%)	4 (33%)
**gender**	Female	33 (69%)	14 (67%)	9 (60%)	8 (67%)
	Male	15 (31%)	7 (33%)	6 (40%)	4 (33%)
**Referral source**	Facebook	15 (32%)	8 (38%)	8 (54%)	7 (59%)
	Twitter	8 (17%)	2 (9.5%)	0 (0%)	0 (0%)
	Flyer	9 (19%)	2 (9.5%)	1 (6.5%)	1 (8%)
	Centre website/magazine	4 (8%)	2 (9.5%)	1 (6.5%)	0 (0%)
	Cancer information centre	4 (8%)	4 (19%)	3 (20%)	2 (17%)
	Support group	4 (8%)	2 (9.5%)	1 (6.5%)	1 (8%)
	Newspaper/radio	3 (6%)	1 (5%)	1 (6.5%)	1 (8%)
	Volunteer website	1 (2%)	0 (0%)	0 (0%)	0 (0%)
**Cancer diagnosis**	Breast	15 (32%)	8 (38%)	4 (27%)	3 (25%)
	Prostate	2 (4%)	2 (9.5%)	2 (13%)	1 (8%)
	Lung	0 (0%)	0 (0%)	0 (0%)	0 (0%)
	Colorectal	3 (6%)	2 (9.5%)	1 (7%)	1 (8%)
	Gynaecological	7 (15%)	2 (9.5%)	2 (13%)	1 (8%)
	Other cancers	14 (29%)	7 (33.5%)	6 (40%)	6 (50%)
	Unknown	6 (12%)			
	No cancer	1 (2%)			
**Total**		**48**	**21**	**15**	**12**

Social media proved an effective recruitment mode, with many potential peer mentors identified using such methods (Table 2), usually mediated by the cancer centre media channels. Those who were fully trained were particularly likely to have discovered the study via social media:

I’ve seen in Facebook actually, I was looking somewhere, researching, and looking and I seen the advertisement. M2

Macmillan had shared it on a Facebook post, which I then "liked" and followed through and made direct contact with you. M6

I found out about it on [name of local radio station]. M8

Like, say I’m a friend of [Cancer Centre A], so it’d come through there and we just spotted it. Now, that’s where that come from and then I phoned. M9

Enquiries continued to be received throughout the study, an effect of media led recruitment, and which led to disappointment from some who contacted the team too late for training.

### Motivation for Becoming a Peer Mentor

Potential peer mentors wanted to ‘give something back’:

I just felt that I wanted to give something back and I thought it was something achievable that I could do that, you know, rather than committing myself to working in a hospice every week or something. M2

People volunteered as peer mentors typically because of their own experiences, knowing how they would have valued such support during their own cancer experiences:

Because I wished I had had a buddy at the beginning, when I started. M4

…it's quite daunting when you're going through treatment and it's nice to have someone you can refer to and ask specific questions. Because I think people very much are so grateful to the health professionals for the care that they receive and they don’t want to be troubled so they feel like they're being mithered if they're saying, "What about this?" or, "What about that?" Whereas, if you have someone unofficial you can go to or not directly connected with your treatment, I think they're more likely to open up. M6

Potential mentors therefore brought their own wishes and experiences to the training. This meant the training drew heavily from sharing of these experiences and considering how these could be used, or not, to support others.

### Timing and Length of Training

Four 2-day training sessions were held. We had anticipated holding fewer training sessions with more participants per session, but availability of sufficient participants to make a session viable proved challenging. Many people who did not proceed through the training were either unavailable for planned initial training days (*n* = 10) or too unwell (*n* = 10). Some availability issues were because we were holding training across two city locations approximately 30 miles apart, but potential peer mentors came from a broader geographical catchment area and wanted to attend local training. Participants noted the advantages of a larger group, with training completed over a shorter period:

A bigger group, a bit more knowledge and a bit know the different people, having different cancer and yes, it was really, really good that, yeah. I wish I could continue with them, but we didn’t manage [participant had to attend a different day 2 training]. If it had a bit more training, maybe, like two times a week, a few weeks, you could just maybe a bit more know each other. M3*.*

People appeared to appreciate a day for training, as this gave time to both get to know other potential mentors and give time for reflection and consideration on content:

I didn’t mind it, because you had a decent break at lunchtime. I think that lunch time gave everyone an opportunity to talk to each other, so I think it would be too much to condense it, because you would just sit there and think wow, I have got an hour and a half and I have got to take all this in. So, I thought it worked really well. M4

The written materials were perceived as helpful, and an aide-memoire if required:

It’s lodged somewhere in your mind and I suppose that’s what the idea of the folder, if I’d have sort of sat down and looked through it again. M2

Of those who enquired about being a peer mentor during the study recruitment period, 25% eventually completed training as peer mentors.

### Perceptions of Training

Participants valued the 2-day training programme. Day 1 focused on core training, and day 2 presented a novel training module based on how to cope well with advanced cancer based on our earlier research.

Potential peer mentors shared concerns about their perceptions of the training and the role:

I was a bit apprehensive at parts when we were doing the training, for me personally, not anyone else. I was worried about not having had chemo, which I do think to me would have made a big hurdle for someone who is maybe further down the line, has had chemo, and I wouldn’t think they could relate to me because I didn’t. That was one problem, one thing I had really at the back of my mind. But I was looking forward to doing it, you know. M8

Mentors were concerned that they needed to be ‘similar’ to those that they may eventually be paired with or that their own unique experiences may render them unsuitable to be peer mentors. These concerns were mitigated not only through the formal training but also through the supportive connections potential peer mentors made through the training process:

Because you know like [name 1] and [name 2] and I have become friends. I mean, we see each other, not regularly but we meet up and we go out for lunch, and we’d all had a talk about this before in the training, how it was going to affect us. So we said, “Right, now we’re going to really give it a go. We’ll take the first one. If it doesn’t work – and we’ll back each other up, and that’s how we’ll work it.” M8

Mentors appeared to appreciate the mentor-led, interactive and informal nature of the training, supported by written materials. Whilst they expressed concern about role play, they recognized why it might be important and how it contributed to training:

Yeah, it was okay, that was the day we did like role play and things. I suppose it was useful, I don’t particularly like anything like that even from when I was at school, it’s not my sort of thing but I know that you have to do it in these sort of…Yeah, it’s just not something I’m used to doing, I just felt a bit awkward with it but, yeah. And I think from what I can remember I think we ended up actually not being role play almost talking as if it was real. M2

Data from the feasibility study are reported elsewhere, but it is worthwhile noting that no incidents or adverse events occurred during the study.

## Discussion and Conclusions

### Discussion

These data demonstrate that it is feasible to recruit and train people to be peer mentors for those with advanced cancer. Effective ways of reaching out to people include via social and traditional media forms, rather than relying on health or social care professionals to identify potential peer mentors. Approximately a quarter of those who are interested may eventually be available as peer mentors. Availability and timing of training is a critically important facilitating factor. Attrition due to the potential peer mentor’s ill health has to be anticipated and planned for.

These are important and novel data for those interested in peer mentor intervention, not just in the context of research. Knowledge about how and from where peer mentors are recruited is extremely scanty; most research either focuses on the recipients of the peer mentor intervention or their training, not recruitment. Thus, a recent meta-synthesis identified data focused on the content of training, but not recruitment [40], and even a study on the experience of peer supporters does not identify from where they were recruited [17]. No data were given on this issue in the most recent peer mentor trial published [43]. On the few occasions where there is reporting, it is minimal, as in this example: ‘Peer advocates were recruited from the practices of UWBC clinicians and received in-person training on six dimensions of peer advocacy’ [29]. It is likely that many studies, who do not report on the source of peer mentors, similarly recruit from ‘known’ sources such as clinics. We have demonstrated it is possible to recruit more broadly and the effectiveness of social media in this. Social media has been identified as a route for recruiting collaborators, patients and parents, but not providers of an intervention such as in peer mentoring [3, 20, 27].

Recruiting peer mentors is feasible. Individuals are open to and accept training and the bureaucratic but important elements such as police checks. This is important, as some are anxious or fearful about the role or viability of peer mentors [23, 41]. Peer mentoring, however, is not for everyone who might indicate an interest. Attrition due to incompatibility with the role, availability, time commitment or own illness must be taken into account. It was not possible to recruit as broad a range of participants as might be required – with a preponderance of females with breast cancer. This is typical in peer support studies but may be problematic if matching is desired on gender or diagnostic grounds [45].

### Strengths and Limitations of the Study

It was necessary, given the mode of recruitment of peer mentors, to accept their own appraisal of their diagnosis and prognosis as we did not have access to their clinical notes to verify this information. In future studies, it may be helpful to seek participant approval to request this information.

### Conclusions

We have demonstrated it is feasible to recruit peer mentors to support people with advanced cancer who themselves have recent or ongoing diagnoses of cancer*.* It is important that this recruitment and training is responsive and flexible, taking account of the vulnerabilities of those who may volunteer to be peer mentors. Plans must include contingencies for large proportions of those who volunteer to be unable to fulfil this role, often due to their own ill health. Our data reported here on source, flow and attrition of peer mentors should enable people to consider these issues in a more considered way. We summarize the transferrable learning from this study in Table 3.

**Table 3:** Key learning points on peer mentor recruitment

Source of recruitment	A broad approach is possible and feasible. Consider traditional forms of recruitment such as via health care services, but also through traditional media (newspaper/radio) and social media (Facebook/Twitter). Social media channels mediated by trusted people (e.g. healthcare channels) may be particularly effective
	Flexible approaches to recruitment mean that a ‘stop’ to information about the study is challenging, and enquiries must be anticipated throughout the life of the study
Availability/training	Set dates/times in advance for training, and promote these through the recruitment channels. This may reduce enquiries from those who will not be able to attend training
Geographical location	The peer mentor ‘service’ should be geographically located, if to be provided face to face. This facilitates attendance at geographically convenient training locations and a geographical basis to matching patients and mentors
Attrition	Approximately ¼ of the people who enquired completed training. Setting a closer time period between day 1 and day 2 training, and setting dates in advance, may have improved this attrition. Anticipate drop-outs because of ongoing illness or treatment
